# Supplementary material for: Research data management needs assessment for social sciences graduate students: A mixed methods study
Source: PLoS One. 2023 Feb 23;18(2):e0282152. doi: 10.1371/journal.pone.0282152 (PMC9949669; doi:10.1371/journal.pone.0282152)
Supplement: S1 Appendix — (DOCX) [file pone.0282152.s001.docx]

**S1 Appendix. Survey Instrument.**

**Q1. Heard of RDMS at TAMU**

1= Yes

2= No

3= Maybe

**Q2. How did you hear about RDMS (choose all that apply)**

1= Workshops

2= From other faculties, students, or staff

3= Library website

4= n/a

5= other

6=mixed

**Q3. Experience in working with librarian on RDM**

1= Yes

2= No

3= Maybe in the future

4= Probably not

**Q4. Have you ever written a DMP for a grant?**

1= Yes

2= No

3= Maybe

4= Probably not

**Q5. Name of grant for DMP** ________________

**Q6. Familiarity with research data repository**

1= Yes

2= No

3= not sure

**Q7. Have you ever used a research data repository?**

1= Yes

2= No

3= not sure

**Q8. Have you shared your data in a data repository?**

1= Yes

2= No

3= Maybe in the future

4= Probably not

**Q9. Rate your needs for PD in:** (rate from 1-100, rarely needed to greatly needed)

o Writing a DMP

o Data collection and data documentation

o Data analysis and data visualization

o Data preservation and data publication

**Q10. Comfort/confidence level in dealing with RDM** (rate from 1-100, from extremely unconfident to extremely confident)

o Writing a DMP

o Data collection and data documentation

o Data analysis and data visualization

o Data preservation and data publication

**Q11. Preparedness of describing best practices in RDM**

1= very unprepared

2=somewhat unprepared

3= neutral

4= somewhat prepared

5= very prepared

**Q12. Preparedness of describing the research data lifecycle**

1= very unprepared

2=somewhat unprepared

3= neutral

4= somewhat prepared

5= very prepared

**Q13. Preparedness of find and apply discipline-appropriate DM approaches/principles to your research project**

1= very unprepared

2=somewhat unprepared

3= neutral

4= somewhat prepared

5= very prepared
