# Supplementary material for: Research data management needs assessment for social sciences graduate students: A mixed methods study
Source: PLoS One. 2023 Feb 23;18(2):e0282152. doi: 10.1371/journal.pone.0282152 (PMC9949669; doi:10.1371/journal.pone.0282152)
Supplement: Attachment — Submitted filename: Response to Reviewers .docx [file pone.0282152.s002.docx]

| Comment | Response |
| --- | --- |
| Academic editor’s | |
| 1. Please ensure that your manuscript meets PLOS ONE's style requirements, including those for file naming. The PLOS ONE style templates can be found at  <https://journals.plos.org/plosone/s/file?id=wjVg/PLOSOne_formatting_sample_main_body.pdf> and  <https://journals.plos.org/plosone/s/file?id=ba62/PLOSOne_formatting_sample_title_authors_affiliations.pdf> | We formatted our manuscript following the PLOS ONE style templates. |
| 2. Please provide additional details regarding participant consent. In the ethics statement in the Methods and online submission information, please ensure that you have specified what type you obtained (for instance, written or verbal, and if verbal, how it was documented and witnessed). If your study included minors, state whether you obtained consent from parents or guardians. If the need for consent was waived by the ethics committee, please include this information. | We indicated that “all the participants signed the written consent form through Qualtrics before their participation” in the Participants sub-section of the Methods section, the disclosure statement and in the submission system. |
| 3. Peer review at PLOS ONE is not double-blinded (<https://journals.plos.org/plosone/s/editorial-and-peer-review-process>). For this reason, authors should include in the revised manuscript all the information removed for blind review. | Identifying information was added to the manuscript. |
| 4. Thank you for stating in your Funding Statement:  "This work was supported by T3: Texas A&M Triads for Transformation [grant numbers 1762, 2020], Texas A&M University Institutional Funding."  Please provide an amended statement that declares *all* the funding or sources of support (whether external or internal to your organization) received during this study, as detailed online in our guide for authors at <http://journals.plos.org/plosone/s/submit-now>. Please also include the statement “There was no additional external funding received for this study.” in your updated Funding Statement.  Please include your amended Funding Statement within your cover letter. We will change the online submission form on your behalf. | This information was added in the disclosure statement. |
| 5. Thank you for stating the following financial disclosure:  "This work was supported by T3: Texas A&M Triads for Transformation [grant numbers 1762, 2020], Texas A&M University Institutional Funding."  Please state what role the funders took in the study. If the funders had no role, please state: ""The funders had no role in study design, data collection and analysis, decision to publish, or preparation of the manuscript.""  If this statement is not correct you must amend it as needed.  Please include this amended Role of Funder statement in your cover letter; we will change the online submission form on your behalf. | This information was added to our cover letter. |
| 6. In your Data Availability statement, you have not specified where the minimal data set underlying the results described in your manuscript can be found. PLOS defines a study's minimal data set as the underlying data used to reach the conclusions drawn in the manuscript and any additional data required to replicate the reported study findings in their entirety. All PLOS journals require that the minimal data set be made fully available. For more information about our data policy, please see <http://journals.plos.org/plosone/s/data-availability>.  Upon re-submitting your revised manuscript, please upload your study’s minimal underlying data set as either Supporting Information files or to a stable, public repository and include the relevant URLs, DOIs, or accession numbers within your revised cover letter. For a list of acceptable repositories, please see <http://journals.plos.org/plosone/s/data-availability#loc-recommended-repositories>. Any potentially identifying patient information must be fully anonymized.  Important: If there are ethical or legal restrictions to sharing your data publicly, please explain these restrictions in detail. Please see our guidelines for more information on what we consider unacceptable restrictions to publicly sharing data: <http://journals.plos.org/plosone/s/data-availability#loc-unacceptable-data-access-restrictions>. Note that it is not acceptable for the authors to be the sole named individuals responsible for ensuring data access.  We will update your Data Availability statement to reflect the information you provide in your cover letter. | We uploaded our dataset to the Texas Data Repository and shared with the public. The data is available at <https://doi.org/10.18738/T8/YQ1XDX>. |
| 7. We note that you have stated that you will provide repository information for your data at acceptance. Should your manuscript be accepted for publication, we will hold it until you provide the relevant accession numbers or DOIs necessary to access your data. If you wish to make changes to your Data Availability statement, please describe these changes in your cover letter and we will update your Data Availability statement to reflect the information you provide. | The reference for the dataset:  Zhou, Xuan, 2023, "RDM Needs assessment", <https://doi.org/10.18738/T8/YQ1XDX>, Texas Data Repository, V1 |
| 8. Please include captions for your Supporting Information files at the end of your manuscript, and update any in-text citations to match accordingly. Please see our Supporting Information guidelines for more information: <http://journals.plos.org/plosone/s/supporting-information>. | We added the caption for the supporting information at the end of our manuscript. |
| 9. Please review your reference list to ensure that it is complete and correct. If you have cited papers that have been retracted, please include the rationale for doing so in the manuscript text, or remove these references and replace them with relevant current references. Any changes to the reference list should be mentioned in the rebuttal letter that accompanies your revised manuscript. If you need to cite a retracted article, indicate the article’s retracted status in the References list and also include a citation and full reference for the retraction notice. | All references cited in the text are in the reference list. None of the articles have been retracted to our knowledge. |
| Reviewer 1 | |
| It is a well-structured work that helps to understand some of the challenges faced by new researchers who, sometimes, put their investigations at risk due to a lack of these skills. | Thank you. |
| About data - The data should be provided as part of the manuscript or its supporting information, or deposited to a public repository. If there are restrictions on publicly sharing data, those must be specified. | Information about the underlying data was added. |
| Reviewer 2 |  |
| The manuscript addresses a significant problem in the selected area of research. Furthermore, the contents are managed coherently. However, the following observations need redressal: | Thank you. We addressed the comments one by one. |
| 1. Instead of mentioning 'Research Question 1" in the subheading in Results and Discussion, thematic headings would make them more comprehensible. This applies to all the subheadings in this section. | We deleted “research question” from the subheads and added thematic headings for each level. |
| 2. Discussion on the interview data is limited and needs further instances, elaboration, and due support from the reviewed literature. | We elaborated on the discussion of the interview data and better connected the discussion with the literature review. |
| 3. It seems that the data has not properly been triangulated. | This is a mixed-method study. We used interview data to supplement the information we obtained from the survey, but did not triangulate the interview data separately.  We added additional discussion about the interview data. |
| 4. Recommendations for future studies are not specified. | Future research directions were added to the first paragraph of the Conclusion section. |
| 5. The authors might be advised to get the manuscript proofread by an expert in the English language. | A native English speaker proofread and edited the manuscript. |
